# Clinical Course After Transcatheter Aortic Valve Replacement for Takayasu Arteritis–Related Aortic Stenosis and Regurgitation

**DOI:** 10.1016/j.jaccas.2025.105917

**Published:** 2025-11-01

**Authors:** Yuki Tadokoro, Naonori Kawamoto, Kizuku Yamashita, Takashi Kakuta, Kensuke Takagi, Yasuhide Asaumi, Hideaki Kanzaki, Chisato Izumi, Kazuhiro Yamamoto, Satsuki Fukushima

**Affiliations:** a Department of Cardiovascular Surgery, National Cerebral and Cardiovascular Center, Suita, Japan; b Division of Coronary Diseases, National Cerebral and Cardiovascular Center, Suita, Japan; c Department of Heart Failure and Transplantation, National Cerebral and Cardiovascular Center, Suita, Japan; d National Cerebral and Cardiovascular Center Hospital, Suita, Japan

**Keywords:** aortitis, Takayasu arteritis, transcatheter aortic valve replacement

## Abstract

Takayasu arteritis (TAK) is chronic, large-vessel vasculitis that frequently involves the aorta and its major branches, often leading to aortic valve disease. Recently, transcatheter aortic valve replacement (TAVR) has emerged as a less invasive alternative for patients with TAK-induced aortic stenosis who are at high risk for conventional surgical aortic valve procedures. However, long-term data remain limited. We report the clinical courses of 3 patients with TAK who underwent TAVR for aortic stenosis and regurgitation. No major complications were observed during the procedure or hospital stay. The mean follow-up duration after TAVR was 5.7 years. During follow-up, 1 patient experienced a relapse of TAK that led to low cerebral perfusion, heart failure, and subsequent death 7 years after TAVR. Our observations suggest that TAVR may yield acceptable long-term outcomes for high-risk patients with TAK if disease activity can be suppressed, thus preventing heart failure or cerebrovascular events.

## Visual Summary

**Figure:**
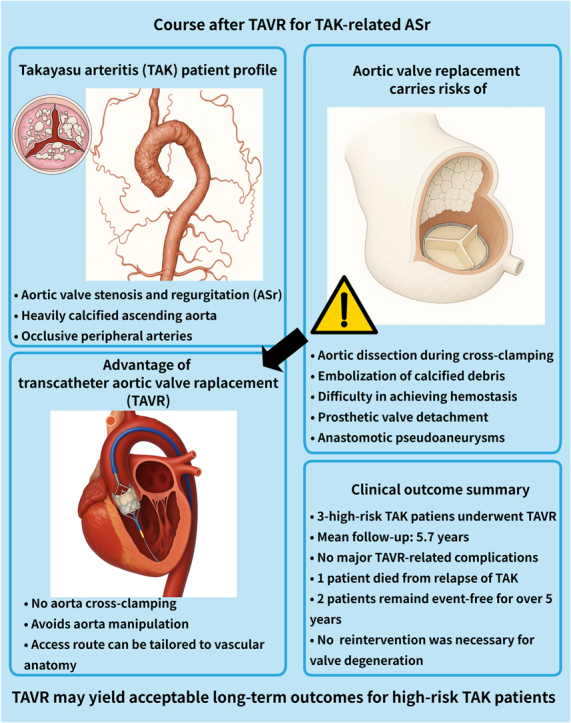

**Visual Summary:**
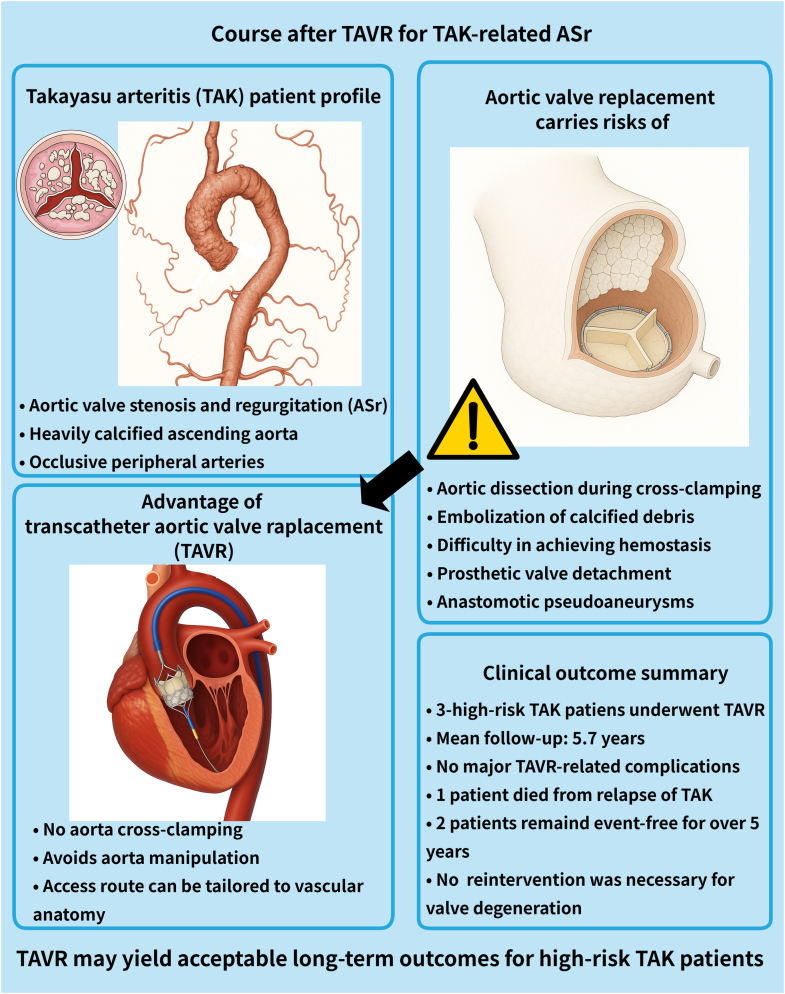
Clinical Course After TAVR for TAK-Related ASr ASr = aortic valve stenosis and regurgitation; TAK = Takayasu arteritis; TAVR = transcatheter aortic valve replacement.

Takayasu arteritis (TAK) is often granulomatous and predominantly affects the aorta and/or its major branches.^1^^,^^2^ It is associated with a high incidence of aortic regurgitation; approximately 23.6% of patients with TAK in Japan have aortic regurgitation.^3^ Among these are rare cases of patients who present with a combination of aortic valve stenosis and regurgitation (ASr).

**Take-Home Messages**

• TAVR may be a safe and feasible alternative to surgical aortic valve replacement in patients with Takayasu arteritis (TAK) with extensive aortic calcification or high surgical risk.
• Avoiding aortic manipulation during TAVR may reduce the risk of valve detachment and pseudoaneurysm formation. By preventing the relapse of TAK with heart failure or cerebrovascular events, TAVR may offer an acceptable long-term outcome for patients with TAK with aortic stenosis and regurgitation.

Aortic valve replacement (AVR) is an option for patients who experience symptoms of heart failure despite medical treatment for TAK. However, it poses a risk of postoperative detachment or the formation of anastomotic pseudoaneurysms.^4^, ^5^, ^6^, ^7^, ^8^ Although transcatheter AVR (TAVR) may be a better alternative than AVR in high-risk patients with TAK-related ASr, early and late outcomes of TAVR in such patients have not been fully reported.^9^^,^^10^ This is the first report to describe the early and late clinical course after TAVR for patients with TAK-related ASr.

## Case Descriptions

### Patient 1

**Figure 1:**
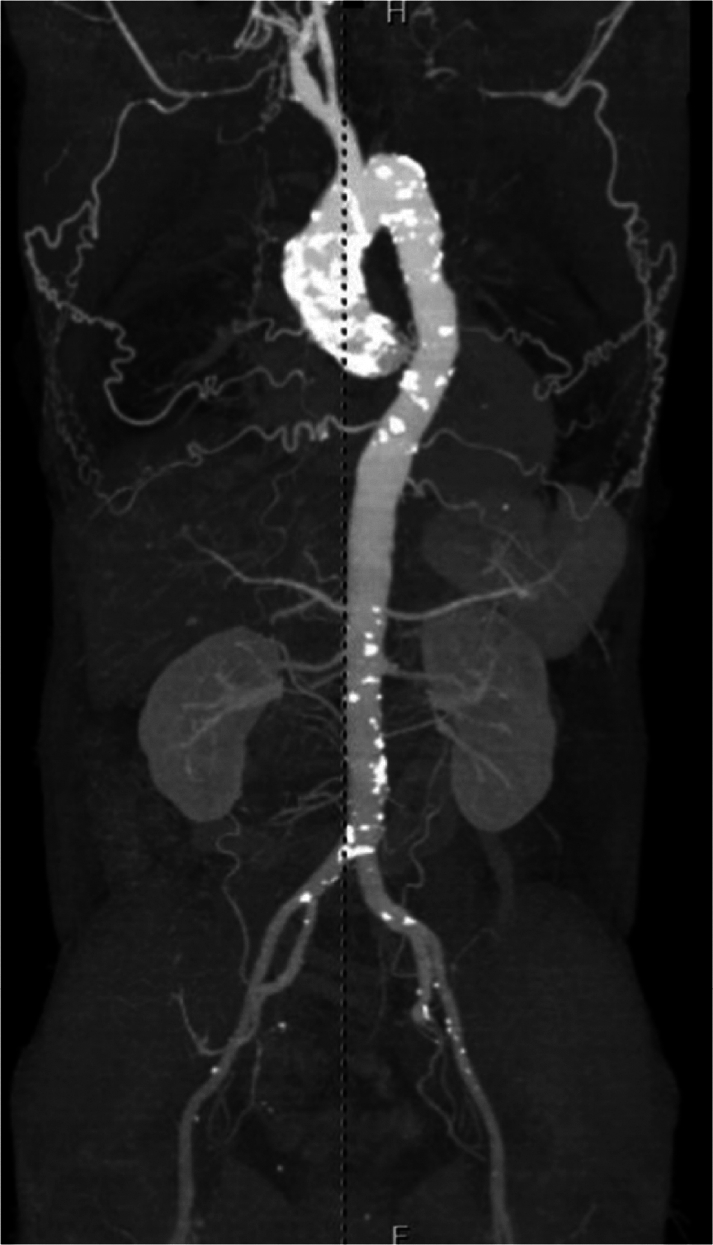
Imaging of Patient 1 Computed tomography revealed circumferential aortic calcification between the aortic root and the descending aorta, left common carotid artery occlusion, and circumferential calcification of both subclavian arteries and the internal thoracic artery.

A 64-year-old woman with a history of hypertension and nephrotic syndrome was referred to our hospital for exertional dyspnea. Echocardiography revealed moderate aortic stenosis (peak jet velocity: 3.5 m/s, mean pressure gradient [mPG]: 24 mm Hg, aortic valve area: 1.01 cm^2^) and severe regurgitation. Computed tomography (CT) revealed circumferential aortic calcification between the aortic root and the descending aorta, left common carotid artery occlusion, and circumferential calcification of both subclavian arteries and the internal thoracic artery (Figure 1).

The diagnosis of TAK was based on the Japanese Circulation Society 2017 guideline on the management of vasculitis syndrome.^2^ However, inflammatory markers were not elevated, and positron emission tomography (PET)-CT revealed no vascular uptake. No concomitant autoimmune diseases were identified. Given the absence of disease activity, immunosuppressive therapy was not initiated. The patient's symptoms of heart failure were mitigated via diuretic therapy; however, after a discussion with the heart valve team, the decision was made to proceed with TAVR.

**Figure 2:**
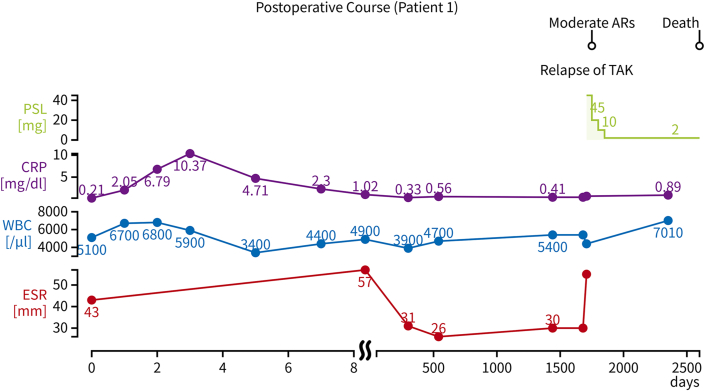
Postoperative Course of Patient 1 Trends in C-reactive protein (CRP) level, erythrocyte sedimentation rate (ESR), white blood cell (WBC) count, and prednisolone (PSL) dosage after transcatheter aortic valve replacement. The patient experienced a relapse of Takayasu arteritis (TAK) at approximately day 1,500, with the subsequent reintroduction of PSL. Moderate aortic regurgitation (AR) developed, and the patient died 7 years 3 months postoperatively.

After valvuloplasty of the aortic valve with a 20-mm balloon, TAVR was performed by using a 23-mm Sapien XT valve (Edwards Lifesciences) via a transfemoral approach, and no vascular complications occurred. Postoperative echocardiography revealed no paravalvular leakage or stenosis (peak jet velocity: 2.1 m/s, mPG: 10.8 mm Hg, effective orifice area [EOA]: 1.5 cm^2^). The postoperative course was uneventful, and the patient was discharged on postoperative day 7 without immunosuppressive therapy. Single-antiplatelet therapy was initiated on the second day after TAVR and continued for 6 months postoperatively. No percutaneous coronary intervention procedures were required during follow-up. During the >5-year follow-up, echocardiography showed no progression of structural valve deterioration (SVD) (peak jet velocity: 3.2 m/s, mPG: 21 mm Hg, EOA: 1.1 cm^2^). However, the patient experienced prerenal kidney dysfunction and exacerbation of her symptoms of cerebral ischemia, including dizziness and lightheadedness. Doppler ultrasonography revealed stenosis of the right renal artery and occlusion of the right external carotid artery. Based on these observations, she was diagnosed with a TAK relapse, and corticosteroid therapy with 45 mg prednisolone was initiated. At the 7-year postoperative follow-up, the patient had been taking 80 mg telmisartan, 80 mg nifedipine, 8 mg torasemide, 40 mg furosemide, 1 mg trichlormethiazide, and 2 mg prednisolone. However, her dyspnea had worsened, and her B-type natriuretic peptide levels had increased to 1,612 pg/mL. An attempt was made to intensify the diuretic and cardioprotective medications; however, her symptoms of cerebral ischemia (eg, dizziness and lightheadedness) worsened. PET-CT demonstrated an increased uptake in the brachiocephalic artery and right common carotid artery. The patient's heart failure worsened before azathioprine could be initiated to control the disease activity, and she experienced cardiac arrest in the emergency room. She subsequently developed hypoxic encephalopathy. The patient died 7 years 3 months after the surgery. Her postoperative course and trends in her C-reactive protein level, erythrocyte sedimentation rate, white blood cell count, and prednisolone dosage after TAVR are illustrated in Figure 2.

### Patient 2

A 70-year-old woman with TAK underwent coronary artery bypass grafting (CABG) at the age of 34 for coronary artery stenosis. No concomitant autoimmune diseases were diagnosed in this patient. Follow-up echocardiography demonstrated progressive worsening of aortic stenosis. Therefore, she was referred to our hospital for surgical management.

**Figure 3:**
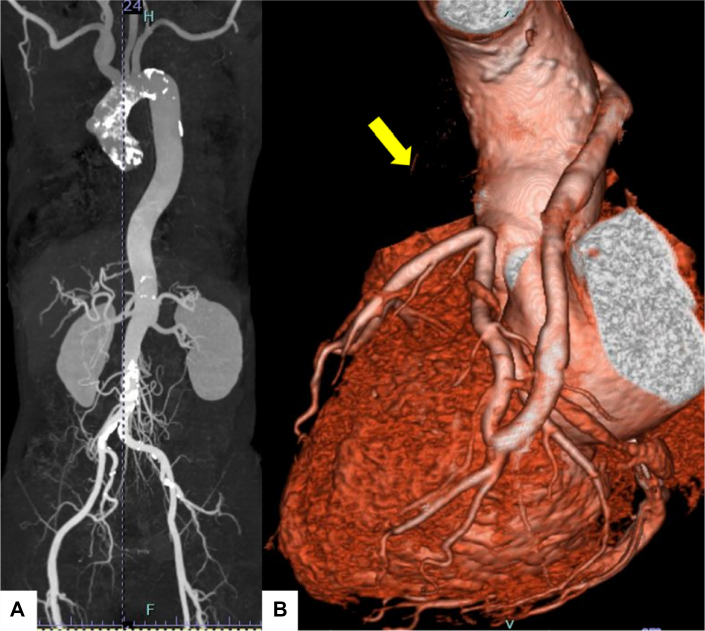
Imaging of Patient 2 (A) Computed tomography revealed circumferential aortic calcification extending from the aortic root to the descending and abdominal aorta, as well as 50% stenosis of the left common carotid artery. (B) Coronary computed tomography angiography revealed patency of the saphenous vein graph (SVG) in the obtuse marginal branch, whereas the SVG in the left anterior descending artery was occluded (yellow arrow).

Echocardiography revealed severe aortic stenosis (peak jet velocity: 5.1 m/s, mPG: 57 mm Hg, aortic valve area: 0.73 cm^2^) and moderate regurgitation. CT revealed circumferential aortic calcification extending from the aortic root to the descending and abdominal aorta, as well as 50% stenosis of the left common carotid artery (Figure 3). Myocardial perfusion imaging revealed no signs of myocardial ischemia. The disease activity of the patient's TAK had been well controlled with a maintenance dose of prednisolone (5 mg daily). After discussion with the heart valve team, the decision was made to proceed with TAVR.

TAVR was performed using a 20-mm Sapien 3 valve (Edwards Lifesciences) via a transfemoral approach, and no vascular complications occurred. Postoperative echocardiography revealed no noteworthy paravalvular leakage or SVD (peak jet velocity: 3.4 m/s, mPG: 26 mm Hg, EOA: 1.02 cm^2^). The postoperative course was uneventful, and the patient was discharged on postoperative day 7. The disease activity of the patient's TAK remained stable with the maintenance dose of prednisolone (5 mg daily); Single-antiplatelet therapy was initiated on the second day after TAVR and continued for 6 months postoperatively. No percutaneous coronary intervention procedures were required during follow-up.

**Figure 4:**
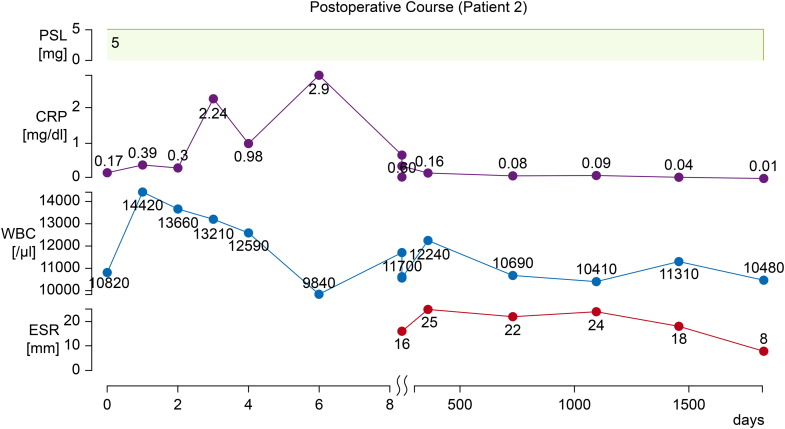
Postoperative Course of Patient 2 Trends in C-reactive protein (CRP) level, erythrocyte sedimentation rate (ESR), white blood cell (WBC) count, and prednisolone (PSL) dosage after transcatheter aortic valve replacement. Inflammatory markers remained stable under low-dose PSL, with no evidence of Takayasu arteritis relapse throughout the follow-up period.

At the 5-year postoperative follow-up, PET-CT revealed no noteworthy uptake in the major arteries, indicating adequate control of TAK. Echocardiography revealed elevation of the pressure gradient (peak jet velocity: 4.4 m/s, mPG: 40 mm Hg, EOA: 1.1 cm^2^) and trivial transvalvular aortic regurgitation. The patient remained free of heart failure symptoms and continued using enalapril (5 mg) and prednisolone (5 mg) (Figure 4).

### Patient 3

A 68-year-old woman underwent CABG at the age of 61 for coronary artery stenosis. She had undergone routine follow-up with transthoracic echocardiography, which demonstrated progressive worsening of aortic stenosis, thus she was referred to our hospital for surgical management.

**Figure 5:**
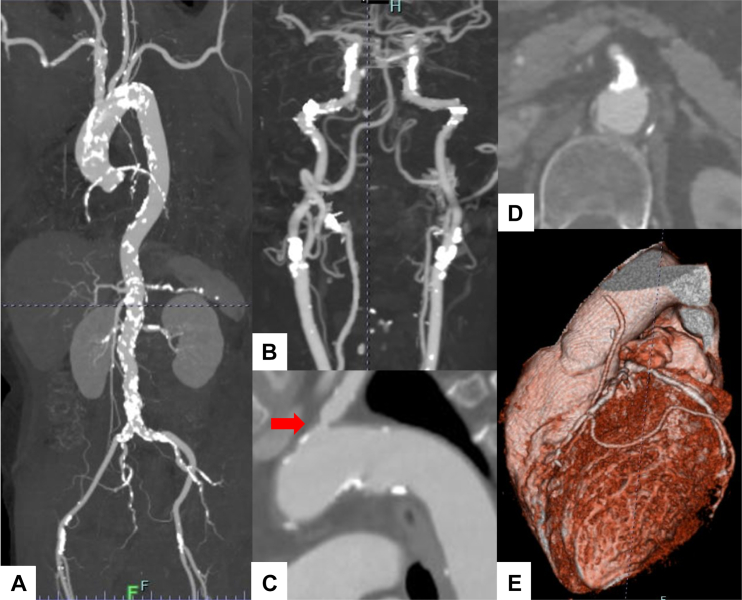
Imaging of Patient 3 Computed tomography revealed widespread calcified stenoses affecting (A and B) the bilateral common and internal carotid arteries, (C) left subclavian artery (red arrow), and (D) superior mesenteric artery. (E) Coronary computed tomography angiography revealed the patency of the left internal thoracic artery graft in the left anterior descending and posterolateral branches.

Echocardiography revealed severe aortic stenosis (peak jet velocity: 4.5 m/s, mPG: 82 mm Hg, aortic valve area: 0.59 cm^2^) and moderate regurgitation. CT revealed widespread calcified stenoses affecting the bilateral common and internal carotid arteries, the left subclavian artery, the superior mesenteric artery, the left renal artery, and both femoral arteries (Figure 5). TAK was diagnosed based on the Japanese Circulation Society guideline on the management of vasculitis syndrome.^2^ However, the patient's inflammatory markers were not elevated, and PET-CT revealed no vascular uptake. No concomitant autoimmune diseases were identified. Given the absence of disease activity, immunosuppressive therapy was not initiated. After discussion with the heart valve team, surgical AVR was considered high risk, and transapical TAVR was planned because of peripheral artery narrowing.

TAVR was performed using a 23-mm Sapien 3 valve via a transapical approach; no bleeding events occurred. Postoperative echocardiography revealed no paravalvular leakage or SVD (peak jet velocity: 1.5 m/s, mPG: 5 mm Hg). The postoperative course was uneventful, and the patient was discharged on postoperative day 7. The disease activity of TAK remained stable without immunosuppressive therapy. Post-TAVR treatment consisted solely of single-antiplatelet therapy for 6 months, without any anticoagulants. No percutaneous coronary interventions were required during follow-up.

**Figure 6:**
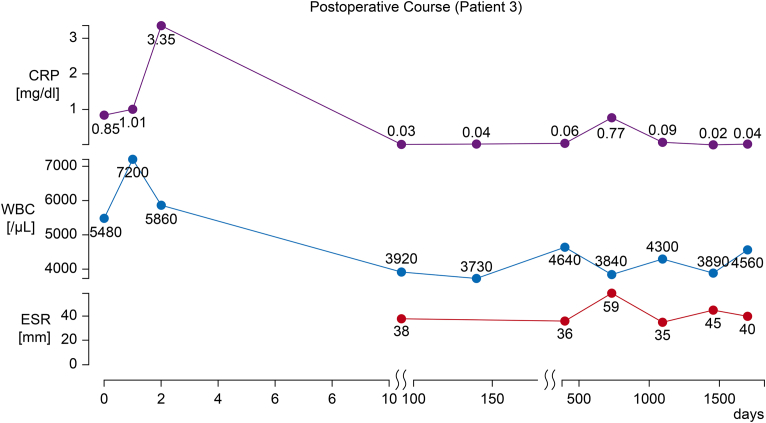
Postoperative Course of Patient 3 Trends in C-reactive protein (CRP) level, erythrocyte sedimentation rate (ESR), and white blood cell (WBC) count after transcatheter aortic valve replacement. CRP levels remained low during follow-up, whereas the ESR fluctuated mildly.

At the 5-year postoperative follow-up, echocardiography showed no noteworthy SVD (peak jet velocity: 2.1 m/s, mPG: 9 mm Hg, EOA: 1.28 cm^2^). Although PET-CT revealed no noteworthy vascular uptake, carotid ultrasonography suggested that the stenosis had progressed. The patient had not developed heart failure symptoms by the time of reporting, however close follow-up with medical therapy was ongoing, including 5 mg carvedilol (Figure 6).

## Discussion

This case series demonstrates 3 major points. First, TAVR demonstrated safer surgical outcomes, with no major complications and shorter hospital stays compared with AVR for patients with TAK, even if they had a heavily calcified ascending aorta and occlusive peripheral arteries. As seen in these 3 patients, in the presence of severe aortic calcification, AVR may pose considerable risks, including aortic dissection during aortic cross-clamping, embolization of calcified debris leading to stroke, and difficulty in achieving hemostasis during suturing. In such cases, TAVR, which avoids direct manipulation of the aorta, is a valuable alternative to consider. However, in TAK, major arteries are often affected by calcification. In the present cases, the transfemoral approach was used in 2 patients and the transapical approach in the other patient, and no vascular complications were observed. These observations highlight the importance of careful selection of the access routes on a case-by-case basis.

The second point is that late prosthetic valve detachment and anastomotic pseudoaneurysms, potentially lethal complications after surgical AVR, are technically improbable events after TAVR. The reported incidence of such complications after AVR in patients with TAK is 6% to 13%.^4^, ^5^, ^6^, ^7^, ^8^ The inflammation associated with TAK can contribute to prosthetic valve detachment and the development of anastomotic pseudoaneurysms.^4^ Because TAVR does not involve aortic manipulation and yields less of an inflammatory response than surgical AVR, we were surprised that no prosthetic valve instability was observed during long-term follow-up.

In 2 of our cases, mild progressive degeneration of the prosthetic valve was observed; however, reoperation for prosthetic valve degeneration had not been required by the time of reporting.

The third point is that well-controlled disease activity of TAK may be favorable for long-term survival after TAVR. The 2 patients in this study with controlled disease activity of TAK survived beyond 5 years without symptoms of heart failure or cerebrovascular events. However, patient 1, who had a TAK relapse, experienced symptoms of heart failure. Although her medical therapy was initially escalated, it eventually had to be discontinued because of impaired cerebral perfusion. Yamashita et al^10^ reported the surgical outcomes after TAVR and CABG in a patient with TAK. That patient died 7 years later from impaired cerebral perfusion due to uncontrolled disease activity despite receiving immunosuppressive therapy with prednisolone and tocilizumab along with cardioprotective medications. Considering the severe calcification of the thoracic aortic wall, the feasibility of performing TAVR-in-TAVR in future, and the potential for coronary events, we opted for the Edwards Sapien series of valves in all 3 of our cases.

Taken together, TAVR may yield acceptable long-term outcomes in high-risk patients with TAK if the disease activity can be suppressed, thereby preventing heart failure or cerebrovascular events. After TAVR in patients with TAK with ASr, cerebrovascular assessments and echocardiography should be incorporated into regular checkups.

## Conclusions

In terms of procedural safety and postoperative hemodynamics, TAVR may be a viable alternative to surgical AVR for patients with TAK, particularly for those with extensive aortic calcification or a high surgical risk. Better long-term clinical outcomes may depend on the adequate control of TAK disease activity, which suggests that the careful monitoring of disease activity as well as cerebrovascular evaluations are essential during follow-up.

### Data Availability

The data used in this study will be shared upon reasonable request to the corresponding author with permission from the ethics committee.

## Funding Support and Author Disclosures

Institutional review board number: M30-026-10. The authors have reported that they have no relationships relevant to the contents of this paper to disclose.
